# *Trichinella spiralis* Calreticulin Binds Human Complement C1q As an Immune Evasion Strategy

**DOI:** 10.3389/fimmu.2017.00636

**Published:** 2017-05-31

**Authors:** Limei Zhao, Shuai Shao, Yi Chen, Ximeng Sun, Ran Sun, Jingjing Huang, Bin Zhan, Xinping Zhu

**Affiliations:** ^1^Department of Medical Microbiology and Parasitology, School of Basic Medical Sciences, Capital Medical University, Beijing, China; ^2^Research Centre of Microbiome, Capital Medical University, Beijing, China; ^3^Department of Pediatrics, National School of Tropical Medicine, Baylor College of Medicine, Houston, TX, United States

**Keywords:** *Trichinella spiralis*, calreticulin, complement C1q, classical complement activation, complement attack, macrophage, immune evasion

## Abstract

As a multicellular parasitic nematode, *Trichinella spiralis* regulates host immune responses by producing a variety of immunomodulatory molecules to escape from host immune attack, but the mechanisms underlying the immune evasion are not well understood. Here, we identified that *T. spiralis* calreticulin (*Ts*-CRT), a Ca^2+^-binding protein, facilitated *T. spiralis* immune evasion by interacting with the first component of human classical complement pathway, C1q. In the present study, *Ts*-CRT was found to be expressed on the surface of different developmental stages of *T. spiralis* as well as in the secreted products of adult and muscle larval worms. Functional analysis identified that *Ts*-CRT was able to bind to human C1q, resulting in the inhibition of C1q-initiated complement classical activation pathway reflected by reduced C4/C3 generation and C1q-dependent lysis of antibody-sensitized sheep erythrocytes. Moreover, recombinant *Ts*-CRT (r*Ts*-CRT) binding to C1q suppressed C1q-induced THP-1-derived macrophages chemotaxis and reduced monocyte–macrophages release of reactive oxygen intermediates (ROIs). Blocking *Ts*-CRT on the surface of newborn larvae (NBL) of *T. spiralis* with anti-*Ts*-CRT antibody increased the C1q-mediated adherence of monocyte–macrophages to larvae and impaired larval infectivity. All of these results suggest that *T. spiralis*-expressed *Ts*-CRT plays crucial roles in *T. spiralis* immune evasion and survival in host mostly by directly binding to host complement C1q, which not only reduces C1q-mediated activation of classical complement pathway but also inhibits the C1q-induced non-complement activation of macrophages.

## Introduction

Trichinellosis is a serious food-borne parasitic zoonosis caused by eating raw or undercooked meat contaminated with *Trichinella spiralis* larvae cysts (1) and results in the manifestation of fever, facial edema, severe diarrhea, myositis, or even death (2). More than 11 million people are estimated to be infected with *T. spiralis* worldwide (3). Trichinellosis is considered as an emerging or re-emerging disease due to the increased consumption of meat (1, 3). During the host infection by *T. spiralis*, muscle larvae (ML) are released from cysts and then develop into adult worms in small intestine, where female worms produce newborn larvae (NBL). NBL penetrate intestine and disseminate through blood circulation to muscles, where they develop into ML. *T. spiralis* in the three different developmental stages are all exposed to host immune system (4) and develop comprehensive immune evasion strategies in the long process of evolution to survive in the hostile immune environment within host. Understanding the parasite’s immune evasion mechanism would facilitate the development of preventive vaccine or therapeutic drugs against trichinellosis.

The host immune defense network includes innate and adaptive immune system. As a major innate immune component, the complement system is an early barrier for intruding pathogens and is activated directly by pathogens or indirectly by pathogen-bound antibodies. The activation of complement system leads to a cascade of reactions occurring on the surface of pathogens and generates membrane-attack complex (MAC) to attack the invaded pathogens (5). In fact, the pathogen’s immune evasion often targets the host complement system (6). Many studies have demonstrated that some pathogens produce proteins such as human astrovirus coat protein (7), scabies mite inactive serine proteases (8), and *Streptococcus pneumoniae* endopeptidase O (PepO) (9), which can bind human C1q and inhibit the classical pathway of complement activation as a strategy to evade complement attack in the host.

C1q, as the first complement component that can be activated by antibodies (IgG and IgM) bound to antigens/pathogens, plays an initiative and essential role in the activation of classical complement pathway (10). In addition, it also mediates multiple complement-independent functions including binding to C1q receptors on various immune cells to modulate their activities (11). For example, C1q induces chemotaxis of neutrophils (12), eosinophils (13), and macrophages (14) and enhances their adhesion, phagocytosis, or killing abilities to invaded pathogens. Specifically, C1q binds to the C1q receptor on macrophages and triggers the release of both reactive oxygen intermediates (ROIs) and reactive nitrogen intermediates (RNIs), which damage or kill invaded pathogens (15, 16).

Our previous studies demonstrated that *T. spiralis*-expressed paramyosin (*Ts*-Pmy) that bound to C9 and inhibited the formation of MAC to protect the parasite from complement attack (17). Further study revealed that *Ts*-Pmy also bound to C1q to inhibit the initiation of the classical pathway as one of the comprehensive strategies to evade complement activation (18). In addition to *Ts*-Pmy, *T. spiralis* may produce other functional proteins that play important roles in evading complement attack or other immune responses as survival strategies. Recent studies have shown that calreticulin (CRT) in several parasites is involved in immune regulation of host immune system by binding to complement component C1q (19–22).

Calreticulin is a calcium-binding protein conserved in different species of organisms including parasitic helminths (19). It contains globular N-terminal, proline-rich P, and acidic C-terminal domains that participate in multiple functions associated with cell adhesion, calcium storage, and phagocytosis of apoptotic cells (23). Moreover, CRT in certain parasites enables to bind to C1q, which results in inhibiting C1q-dependent complement activity (19, 23), as a major approach to evade host complement attack (20, 21, 24, 25). However, the possible roles of *T. spiralis* CRT (*Ts*-CRT) in the modulation of host immune response, especially in the activation of complement, have not yet been investigated. In this study, *Ts*-CRT was cloned and expressed as a recombinant protein, and its potential ability involved in the immunomodulation was investigated. We found that *Ts*-CRT also bound to human C1q, which resulted in the inhibition of complement classical pathway and C1q-mediated macrophage activities, indicating that *T. spiralis*-expressed CRT plays critical roles in evading host immune responses.

## Materials and Methods

### Animals

Experimental animals were purchased from the Laboratory Animal Services Center of Capital Medical University (Beijing, China) and housed under specific pathogen-free conditions with suitable temperature and humidity. All experimental procedures were approved by the Capital Medical University Animal Care and Use Committee (approval number: 2012-X-108) and were in accordance with the NIH Guidelines for the Care and Use of Laboratory Animals.

### Sera

Normal human serum (NHS) was collected after the signature of an informed consent signed by six healthy volunteers, and the project was approved by the Institutional Review Board (IRB) of Capital Medical University (approval number: 2016SY01). Blood samples were collected by venous puncture by a specialized senior nurse in the Capital Medical University Hospital. Human C1q-deficient serum (C1q-D) was purchased from Merck (Kenilworth, Germany). *T. spiralis*-infected rabbit, swine, and mouse sera were obtained as previously described (26).

### Parasites and Antigen Preparation

*T. spiralis* (ISS 533 strain) was maintained in female ICR mice, and ML were recovered from the muscles of infected mice using a modified pepsin–hydrochloric acid digestion method as previously described (27). Adult worms were isolated from the intestines of infected Wistar rats 108 h after oral larval challenge. NBL were collected from the fertile female adult worms cultured in RPMI 1640 for 48 h at 37°C. Crude somatic extracts of all the stages of *T*. *spiralis* were prepared by homogenizing the worms based on conventional methods (28). The excretory–secretory products of ML (MES) and adult worms (AES) were collected using previously described culture methods (29, 30). Briefly, *T. spiralis* ML were freshly collected and cultured in RPMI 1640 with 0.1% bile swine (Macklin, Shanghai, China) for 48 h at 37°C with 5% CO_2_. The culture supernatants containing MES products were concentrated by centrifugation and buffer exchanged into phosphate-buffered saline (PBS). The AES were obtained with the same methods as for the MES without bile stimulation.

### Cloning, Expression, and Purification of Recombinant *Ts*-CRT (r*Ts*-CRT)

Based on the sequence information of *T. spiralis* calreticulin (*Ts*-CRT) (XM_003371331.1), a pair of specific primers (forward: 5′GCGGATCCGAGCCGACCATTTACCTCAAGGAAAC3′ and reverse: 5′GCCTCGAGCAGCTCTTCTTTAACATT3′) were designed to amplify *Ts*-CRT coding DNA without signal at the 5′-end from adult parasite total cDNA. The amplified *Ts-crt* DNA was subcloned into *E. coli* expression vector pET-28a (Novagen, Darmstadt, Germany). The r*Ts*-CRT was expressed in *E*. *coli* BL21 (DE3) under induction of 0.5 mM IPTG at 37°C for 4 h. The soluble r*Ts*-CRT with His-tags at both N- and C-terminuses was purified by Ni-affinity chromatography (Novagen) following the manufacturer’s protocols. The r*Ts*-CRT purity was analyzed by SDS-PAGE and confirmed by Western blotting with anti-His antibody (TIANGEN, Beijing, China). The r*Ts*-CRT concentration was determined using BCA Protein Assay Kit (Thermo, Waltham, MA, USA). The contaminated endotoxin in purified r*Ts*-CRT was removed using ToxOut Endotoxin Removal Kits (BioVision, San Francisco, CA, USA) and confirmed using the ToxinSensor Endotoxin Detection System (GenScript, Nanjing, China).

### Production of Polyclonal Anti-*Ts*-CRT Antibody

Purified r*Ts*-CRT was used to immunize mice to produce antisera. Mice were subcutaneously immunized with 25 µg of r*Ts*-CRT formulated with an equal volume of adjuvant ISA50v2 (Seppic, Pairs, France). Two boosts were followed by 2-week intervals. One week after the last immunization, the mice sera were collected, and the antibody titer was determined by ELISA. Anti-*Ts*-CRT IgG was purified using HiTrap Protein G HP (GE Healthcare, Uppsala, Sweden) based on the manufacturer’s protocols.

### Calcium-Binding Staining

The calcium-binding property of r*Ts*-CRT was assessed by staining with Stains-all (Sigma, St. Louis, MO, USA), a cationic carbocyanine dye that stains sialoglycoproteins, phosphoproteins, and Ca^2+^-binding proteins blue and all other proteins red (31).

### Expression of Native *Ts*-CRT in Different Stages of *T. spiralis*

The expression of native *Ts*-CRT was analyzed at RNA transcription level by real-time quantitative PCR and protein expression level by Western blotting with mouse anti-*Ts*-CRT antisera at different life stages of *T. spiralis*.

### Real-time Quantitative PCR

Total RNA was extracted from adult worms, ML and NBL using the RNAprep Pure Tissue Kit (TIANGEN) according to the manufacturer’s manual. For total cDNA synthesis, the same amount of total RNA was reverse transcribed using the PrimeScript 1st Strand cDNA Synthesis Kit (Takara, Kusatsu, Japan). All real-time quantitative PCR reactions were performed using TransStrat Top Green qPCR SuperMix (TransGen, Beijing, China) in triplicate. The primers for analyzing *Ts-crt* gene were designed as follows: 5′-CCAAAACATGTCCCAGTACCTG-3′ (forward) and 5′-CTATTGGCCTCAACGCTTCC-3′ (reverse). The primers for GAPDH (the internal expression control) were 5′-TGCTTCTTGCACTACCAATGGCTTAG-3′ (forward) and 5′-ACCAGATGGACCATCGACTGTCTTTT-3′ (reverse). The results of the threshold cycle (Ct) were calculated using 2^−ΔΔCt^ method (32) after being normalized by the house-keeping gene GAPDH; the fold changes of the *Ts-crt* gene expression in adult worms and ML were calculated relative to that in NBL.

### Western Blot

Protein samples (including r*Ts*-CRT; ES products AES and MES; and crude somatic extracts of *T. spiralis* adult worms, ML and NBL) were separated on 12% SDS-PAGE, transferred onto a nitrocellulose membrane (Millipore, MA, USA) and blocked with 5% (w/v) skimmed milk in PBS pH 7.4. Then, the membrane was incubated with mouse anti-*Ts*-CRT sera (1:100,000) or sera from *T. spiralis*-infected rabbits (1:200), mice (1:50), and swine (1:1,000), followed by incubation with IRDye 800CW-conjugated secondary antibody against different species IgG (1:10,000) (LI-COR, Lincoln, NE, USA) and visualized with the Odyssey CLx Infrared Imaging System (LI-COR).

### Immunofluorescence Assay (IFA)

Adult worms and ML of *T. spiralis* were fixed in 4% paraformaldehyde, embedded in paraffin, and longitudinally sectioned. The worm tissue sections were blocked with normal goat serum (ZSGB-BIO, Beijing, China) for 1 h to prevent non-specific binding and were then incubated with anti-*Ts*-CRT mouse sera (1:500) for 1 h. Fixed NBL were permeabilized using 1% Triton X-100 in PBS for 30 min at room temperature and were then blocked and incubated with anti-*Ts*-CRT mouse sera overnight at 4°C. DyLight 488-conjugated goat-anti-mouse IgG (ZSGB-BIO) was used at a dilution of 1:100 for 30 min at 37°C. Worms incubated with normal mice sera under the same conditions served as the negative control. Micrographs were taken with a confocal laser scanning microscopy (Leica, Heidelberg, Germany).

### *Ts*-CRT Binding to Human C1q

#### ELISA

Microtiter plates were coated with different amounts of human C1q (0, 0.2, 0.4, 0.6, 0.8, 1.0, 1.2, and 1.5 µg) (Merck) in 100 μL/well of carbonate buffer (100 mM Na_2_CO_3_/NaHCO_3_, pH 9.6) overnight at 4°C. Control wells were coated with the same amounts of BSA. Following three washes with PBS + 0.05% Tween-20 (PBST), the wells were blocked with 200 µL of 3% BSA in PBS at 37°C for 2 h. After being washed, 0–1.5 µg of r*Ts*-CRT in 100 µL of 20 mM Tris–HCl, pH 7.4, 50 mM NaCl and 1 mM CaCl_2_ were added to each well and incubated for 2 h at 37°C. Then, mouse anti-His mAb (1:10,000, TIANGEN) and HRP-conjugated goat anti-mouse IgG (1:10,000) (BD Biosciences, San Jose, CA, USA) were added and incubated for 1 h at 37°C. After the final washing, the substrate o-phenylendiamine dihydrochloride (OPD, Sigma) was added. The absorbance was measured at 450 nm with an ELISA reader (Thermo).

#### Far Western Blot

Human C1q and BSA (5 µg) were run on 12% SDS-PAGE and transferred onto a nitrocellulose membrane. The membrane was blocked with 3% BSA and then incubated with 5 µg/mL of r*Ts*-CRT in 20 mM Tris–HCl, pH 7.4, 50 mM NaCl, and 1 mM CaCl_2_ at 37°C for 2 h. Binding of r*Ts*-CRT to C1q was detected by anti-His mAb (1:5,000) followed by incubation with IRDye 800CW-conjugated anti-mouse IgG (Li-COR) and then visualized with the Odyssey CLx Infrared Imaging System.

#### Coimmunoprecipitation Assay

To further determine whether C1q binds to non-denatured r*Ts*-CRT or native *Ts*-CRT from ML extracts, Protein G MicroBeads (Miltenyi Biotec, Cologne, Germany) were incubated with 1.5 µg of anti-His mAb + 2 µg r*Ts*-CRT, or anti-*Ts-*CRT antisera (3 µL) + *T. spiralis* ML crude extracts (40 µg) for 30 min on ice; next, 3 µg of human C1q was added at 4°C for 2 h. The beads were washed four times with washing buffer (1% NP40, 50 mM Tris–HCl, 250 mM NaCl, pH 8.0). Finally, 50 µL preheated 1× SDS gel loading buffer was added to the beads to elute the binding protein complex. The eluted proteins were separated by SDS-PAGE and probed with rabbit anti-human C1qA antibodies (1:10,000) (Abcam, Cambridge, UK) and with IRDye 800CW-conjugated goat anti-rabbit IgG (1:20,000) as the secondary antibody.

#### Detection of r*Ts*-CRT-Mediated Inhibition of C3 and C4 Deposition

Plates were coated with human IgM (2 µg/mL) as C1q activator and blocked with 3% BSA in PBS at 37°C for 2 h before the addition of 2 µg of C1q that had been preincubated with different doses of r*Ts*-CRT (0, 1, or 2 µg) or BSA (2 µg). After 1-h incubation, the plates were washed and incubated with C1q-D (1:200) in 1× Veronal buffer (VB, Lonza, Switzerland) containing 0.05% Tween-20 and 0.1% gelatin for 1 h at 37°C. NHS was served as a positive control. After being washed, C4 and C3 depositions were determined using goat anti-human C4 mAb and rabbit anti-human C3b polyclonal antibodies (1:10,000, Abcam), respectively. HRP-conjugated rabbit anti-goat or goat anti-rabbit IgG (BD Biosciences) was used as the secondary antibody. The absorbance was measured at 450 nm with an ELISA reader.

#### Hemolytic Assays

To assess the effect of r*Ts*-CRT on C1q-initiated classical complement activation-mediated hemolysis, 2 µg of C1q was incubated with different amounts of r*Ts*-CRT (0, 1, 2, or 4 µg) or BSA (4 µg) before being added with C1q-D at a dilution of 1:50 in 1× HBSS^++^ (Hank’s balanced salt solution containing 1 mM MgCl_2_, 0.15 mM CaCl_2_, Thermo) for 1 h at 37°C. Subsequently, 100 µL of sheep erythrocytes (SRBC, 10^8^ cells/mL in HBSS^++^) sensitized with rabbit anti-SRBC Abs (Ab-sensitized erythrocytes, Sigma) was added and further incubated at 37°C for 30 min. The reactions were stopped with cold HBSS^++^ containing 10 mM EDTA and centrifuged at 3,000 rpm for 10 min. The amount of released hemoglobin was determined by measuring the absorbance at 412 nm. The hemolytic activity was expressed as the percentage of the total hemolysis in water.

#### Cell Culture

The human leukemia monocytic cell line THP-1 was obtained from China Infrastructure of Cell Line Resource and cultured in RPMI 1640 medium supplemented with 10% FBS at 37°C in 5% CO_2_. THP-1 cells were differentiated into M2 macrophages under treatment with phorbol-12-myristate-13-acetate (PMA) (100 ng/mL, Sigma) for 48 h and with human IL-4 (20 ng/mL) (Prepro Tech, NJ, USA) for an additional 24 h (33).

#### Cell Immunofluorescence Staining

To determine the effect of r*Ts*-CRT on C1q binding to macrophages, THP-1 cells were induced into M2 macrophages using the above methods (33). After being fixed with 4% paraformaldehyde for 20 min at room temperature, the cells were washed with PBS and blocked with normal goat serum for 30 min at room temperature, then C1q (80 µg/mL) preincubated with different concentrations of endotoxin-free r*Ts*-CRT (0, 30, or 60 µg/mL) was added and incubated for 1 h at 37°C. After being washed, the binding of C1q to macrophages was detected using rat anti-C1q mAb (1:100, Abcam) at 4°C overnight and followed by the addition of DyLight 488-labeled goat anti-rat IgG (1:100) (KPL, Milford, MA, USA) for 1 h at 37°C. After being stained, the cells adhered to the cell slides were mounted using ProLong Gold Antifade reagent (ZSGB-BIO) containing DAPI dye for the staining of cell nuclei and analyzed by confocal laser scanning microscopy (Leica).

#### Transwell Chemotaxis Assay

To determine the effect of r*Ts*-CRT on the C1q-induced chemotactic migration of macrophages, a transwell containing an insert (Corning, NY, USA) with an 8-µm-pore membrane was used. THP-1 monocytes-derived macrophages (2 × 10^5^) were added to the upper chamber. The lower chamber was filled with C1q (10 nM) and different amounts of r*Ts*-CRT (0, 3, 6, or 12 µg) to initiate migration with 5% CO_2_ at 37°C for 24 h. After being washed, the cells that had migrated through the membrane and attached to the outside of the membrane were fixed with methanol and stained with Giemsa. The total cells in 10 randomly selected fields of each group were counted under a microscope, based on a previously described method (34). LPS (100 ng/mL) and BSA were used as the positive and negative controls, respectively.

#### Measurement of ROIs Released by C1q-Stimulated Monocytes

To determine whether the binding of r*Ts*-CRT to C1q inhibits C1q-stimulated monocytes to release ROIs including superoxide anion (${\text{O}}_{2}^{-}$) and hydrogen peroxide (H_2_O_2_), a 96-well plate was coated with C1q (1 µg/well) and incubated with different doses of r*Ts*-CRT (0, 2, or 4 µg) or BSA (4 µg) for 2 h at 37°C. After being washed three times with PBS, each well of the plate was inoculated with 5 × 10^4^ THP-1 cells (possessing the C1q receptor) (35, 36), and the incubation was continued for 24 h at 37°C with 5% CO_2_. Cysteamine (Sigma) was used as a ROIs inhibitor. The production of ${\text{O}}_{2}^{-}$ and H_2_O_2_ in the supernatants was measured using a Superoxide Anion Chemoluminescent Detection Kit (Merck) and a Fluorescent Hydrogen Peroxide Assay Kit (Sigma), respectively, according to the manufacturers’ protocols (15).

#### C1q-Mediated Adherence of Monocyte–Macrophages to NBL *In Vitro*

To determine whether *Ts*-CRT expressed on the *T. spiralis* NBL could bind to C1q and block C1q’s function for attracting macrophages, different amounts of purified mouse anti-*Ts*-CRT IgG (0, 20, or 40 µg) were used for incubation with 400 NBL collected from female adult worms in 100 µL RPMI 1640 for 2 h to block native *Ts*-CRT expressed on the worm surface. Purified normal mouse IgG (NMI) and mouse anti-*Ts*87 IgG (each 40 µg) were used as negative and non-relevant antibody controls, respectively. The unbound antibodies were removed by washing with PBS. THP-1-derived monocyte–macrophages that express Fc-γ receptor (FcγRI and FcγRII) (37) were treated with a human Fc receptor-binding inhibitor (eBioscience, San Diego, CA, USA) to block the non-specific FcγR-mediated binding of antibodies. The antibody-treated NBL and the FcγR inhibitor-treated monocyte–macrophages were added at ratio of 1:30 into C1q-coated (1 µg/well) 96-well plates in a final volume of 150 µL. After incubation for 48 h at 37°C, the number of NBL adhered with more than three monocyte–macrophages was counted under an inverted phase-contrast microscope (Leica) as positive worms attached by monocyte–macrophages. The experiments were run in triplicate.

#### Passive Transfer of NBL Treated with Anti-*Ts*-CRT Antibody and Monocyte–Macrophages into Mice

To validate the viability and infectivity of the NBL treated with anti-*Ts*-CRT antibody and monocyte–macrophages as above, each BALB/c mouse was injected through the lateral tail vein with 10,000 treated NBL (5 mice for each group). ML developed from the treated NBL were collected and counted 26 days after injection (17).

### Statistical Analysis

All data were expressed as the mean ± standard deviations and analyzed using a one-way analysis of variance with the GraphPad Prism 5 software (San Diego, CA, USA). *p* < 0.05 was regarded as statistically significant.

## Results

### Expression and Characterization of r*Ts*-CRT

**Figure 1:**
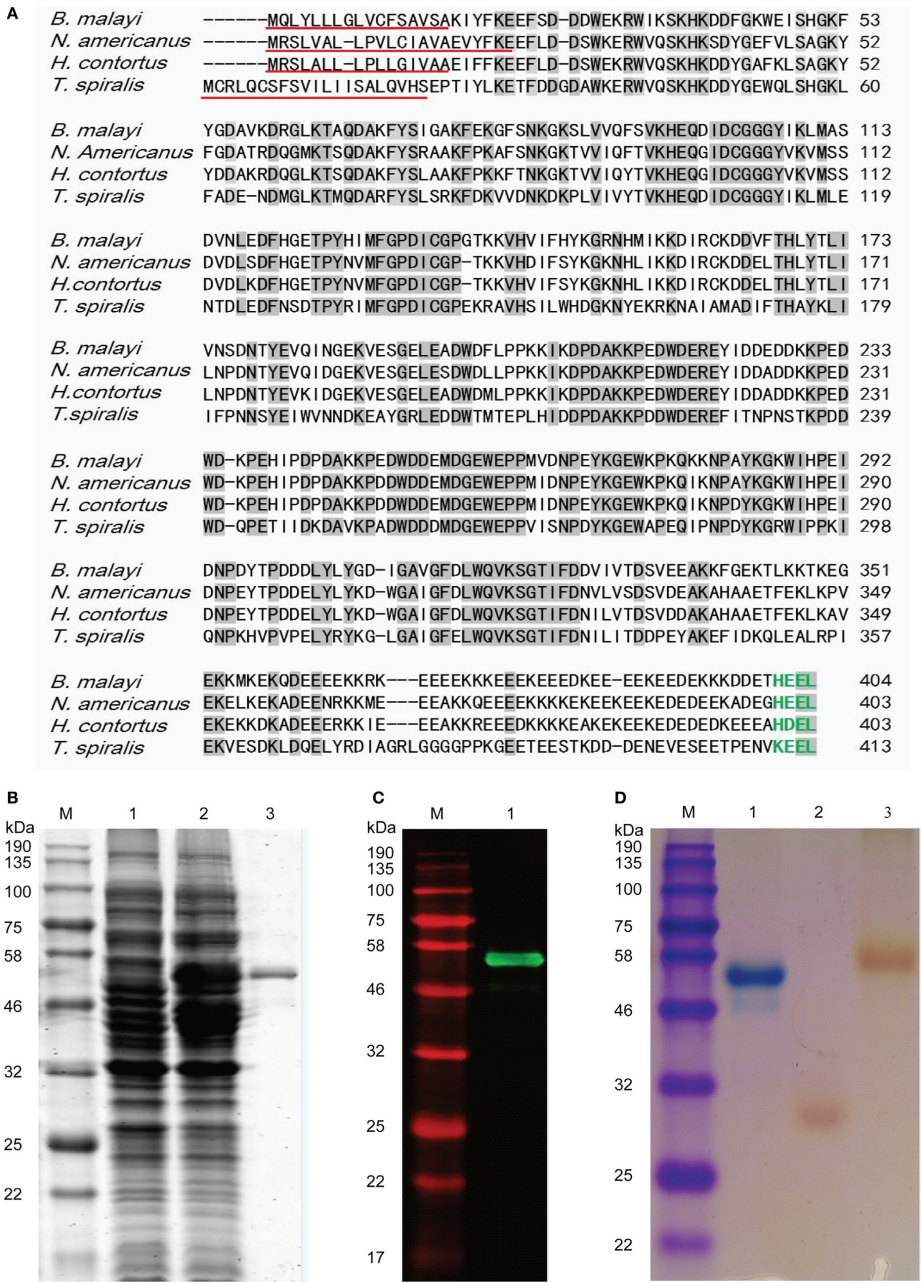
Sequence analysis and characterization of *Trichinella spiralis* calreticulin (*Ts*-CRT). **(A)** Amino acid sequence alignment of *Ts*-CRT (GenBank accession no. XP_003371379.1) with its homologs in other nematodes from *Haemonchus contortus* (CDJ90114.1), *Necator americanus* (CAA07254.1), and *Brugia malayi* (XP_001896170.1) using the ClustalW (http://www.ebi.ac.uk/clustalw) program. The identical amino acid residues in all displayed CRT sequences are indicated with gray shading, the putative signal peptides are underlined in red, and the C-terminal ER retention signal sequences are highlighted in green. **(B)** The r*Ts*-CRT was expressed in *E. coli* and analyzed by SDS-PAGE. Lane 1, uninduced bacteria lysates; Lane 2, IPTG-induced bacteria lysates; Lane 3, purified r*Ts*-CRT protein (500 ng). **(C)** Western blot of purified r*Ts*-CRT (500 ng) probed with anti-His antibody. **(D)** Purified r*Ts*-CRT and other control proteins stained with Stains-all. Lane 1, r*Ts*-CRT stained in blue; Lane 2, r*Ts*-14-3-3 as a *Trichinella spiralis* irrelevant protein control and Lane 3, BSA control stained in red. M, molecular weight marker.

DNA encoding for *Ts*-CRT without the signal peptide was amplified from *T*. *spiralis* adult worm cDNA by PCR using gene-specific primers designed on *Ts-crt* sequence (GenBank accession no. XM_003371331.1). The amplified 1,195 bp DNA fragment was cloned into *E. coli* expression vector pET-28a. The sequence analysis of the *Ts-crt* cloned into pET-28a revealed that the translated amino acid sequence of *Ts*-CRT shares 59, 52, and 51% identity with homologs from *Haemonchus contortus, Necator americanus*, and *Brugia malayi*, respectively (Figure 1A).

Recombinant *Ts*-CRT with His-tags at both N- and C- termini was expressed in *E. coli* BL21 (DE3) as a soluble protein with a size of 53.5 kDa (Figure 1B) corresponding to the predicted molecular mass including the His tags. The purified r*Ts*-CRT was recognized by anti-His antibody (Figure 1C) and stained blue by Stains-all (Figure 1D), indicating that it contains His-tags and is a calcium-binding protein (31). Another recombinant protein r*Ts*-14-3-3 (38) and BSA control protein without calcium-binding activity were stained red by Stains-all.

### Recognition of r*Ts*-CRT by *T. spiralis*-Infected Animal Sera

**Figure 2:**
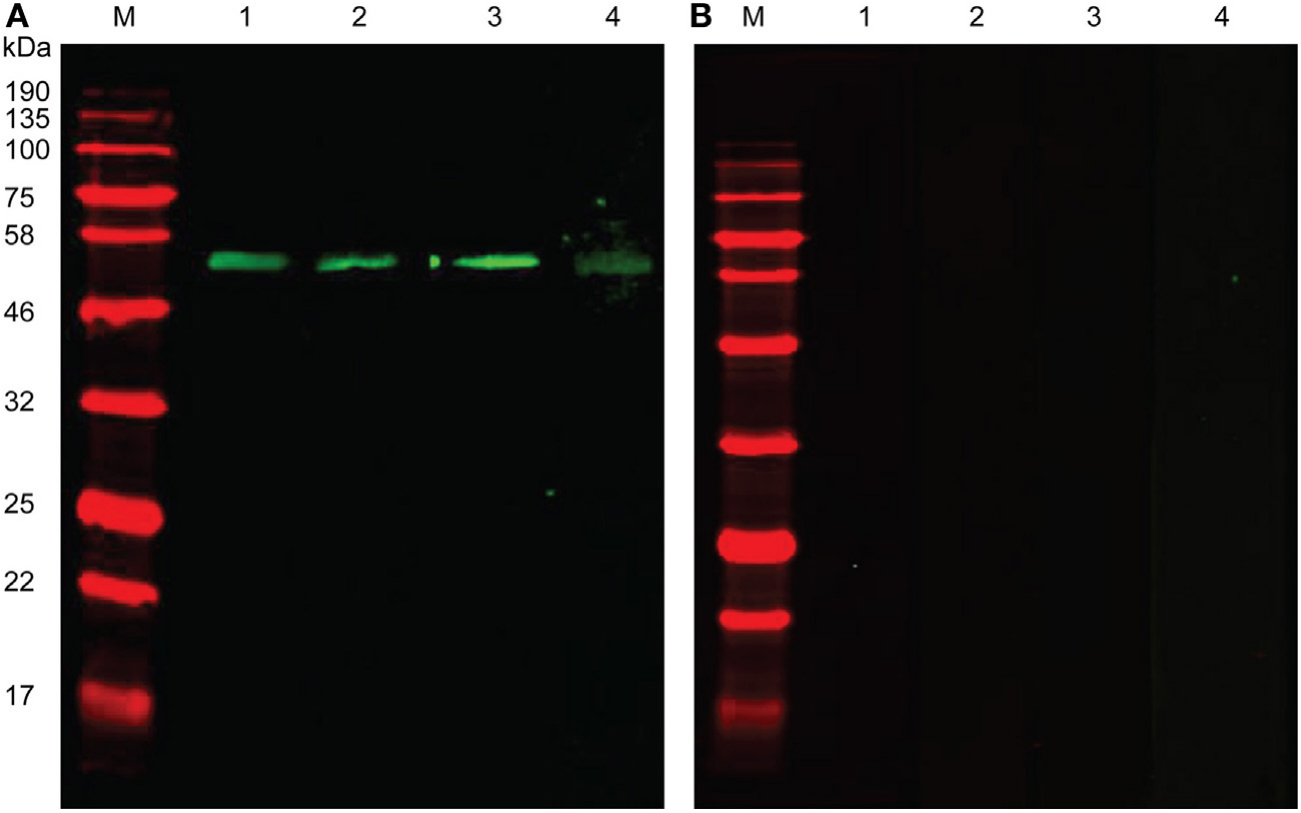
Recognition of recombinant *Trichinella spiralis*-calreticulin (r*Ts*-CRT) by *T. spiralis*-infected animal sera. Western blotting of r*Ts*-CRT was performed with *T. spiralis*-infected animal sera. **(A)** Western blot analysis of r*Ts*-CRT (500 ng) with mouse anti-*Ts*-CRT sera (Lane 1), *T. spiralis*-infected animal sera from rabbits (Lane 2), mice (Lane 3), and swine (Lane 4). **(B)** Western blot with the same amount of r*Ts*-CRT showing no reaction detected by sera from normal mice (Lane 1 and Lane 3), rabbits (Lane 2), and swine (Lane 4). M, molecular weight marker.

Recombinant *Ts*-CRT was used to immunize mice to acquire mouse antisera. Western blot results revealed that *E. coli*-expressed r*Ts*-CRT was strongly recognized by sera from different animals infected with *T. spiralis*, including rabbits, mice, and swine, as well as mouse anti-*Ts*-CRT sera (Figure 2A). No reaction was observed with normal sera from individual animals (Figure 2B). The results indicate that *Ts*-CRT is exposed to the host immune system and is immunogenic during *T. spiralis* infection in different hosts.

### *Ts*-CRT Is Expressed in Different Life Stages of *T*. *spiralis*

**Figure 3:**
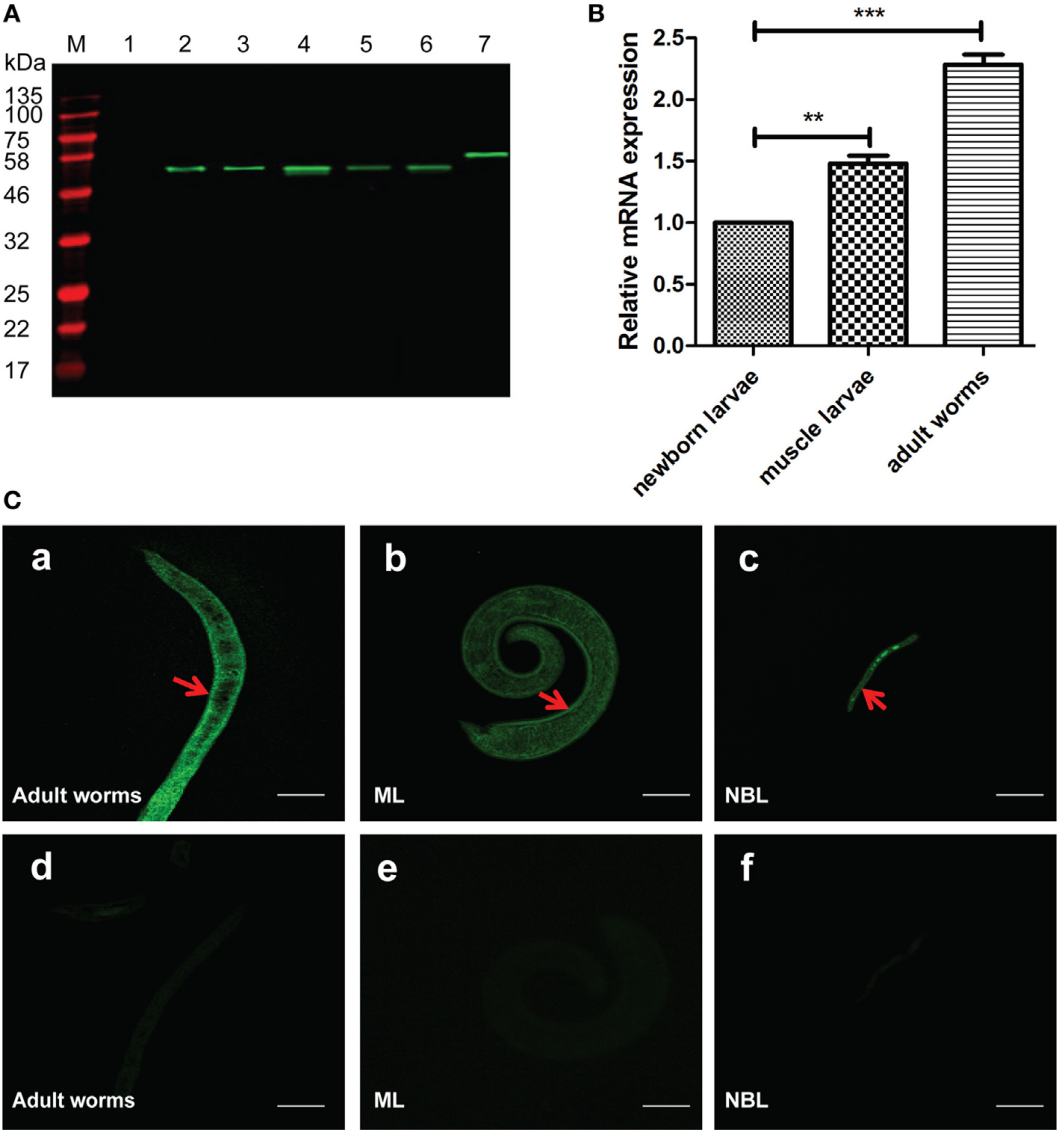
Expression and location of native *Trichinella spiralis* calreticulin (*Ts*-CRT) in the different stages of *T. spiralis*. **(A)** Western blot showing that anti-*Ts*-CRT mouse sera recognized native *Ts*-CRT in all stages of *T. spiralis*, including in the somatic extracts of adult worms (Lane 4), muscle larvae (ML) (Lane 5) and NBL (Lane 6), as well as in the excretory–secretory products of adult worms (AES, Lane 2), ML (MES, Lane 3). Lane 1, recombinant *Ts*87 (500 ng) as an irrelevant protein control; Lane 7, purified recombinant *Ts*-CRT (r*Ts*-CRT) (500 ng); M, molecular weight marker. **(B)** The transcription level of *Ts-crt* mRNA was measured in different developmental stages by real-time quantitative PCR. After being normalized with GAPDH, the gene relative expression level in ML and adult worms was calculated relative to that in NBL (**p < 0.01 and ***p < 0.001). Data are shown as the mean ± SDs for three independent experiments, each experiment was done in triplicates. **(C)** Immunofluorescence assay of *T. spiralis* immunolabeled with mouse anti-*Ts*-CRT antisera. Sections of adult worms (a,d), ML (b,e), and intact NBL (c,f) were incubated with anti-*Ts*-CRT mouse serum (a, b, and c, 1:500) and normal mouse serum (d, e, and f, 1:500), respectively, then probed with DyLight 488-conjugated anti-mouse IgG. The scale bars represent 20 µm. Arrowheads indicate the strong recognition on the parasite surfaces.

Mouse anti-*Ts*-CRT antisera were used in Western blot to analyze the expression of native *Ts*-CRT in different life stages of the parasite. The results demonstrated that anti-*Ts*-CRT mouse sera specifically recognized native *Ts*-CRT in all stages of *T. spiralis* including adult worms, ML, and NBL, as well as in the ES products of adult worms (AES) and muscle larvae (MES) (Figure 3A). There was no reaction to recombinant *Ts*87 (r*Ts*87), another ES protein of *T. spiralis* that was used as an irrelevant protein control (39, 40). The actual molecular weight of native *Ts*-CRT appeared to be approximately 50 kDa, approximately 5 kDa greater than that estimated by the mature protein sequence without the signal peptide (45.5 kDa), probably due to post-translational modification of the protein expressed in the parasite. Real-time quantitative PCR demonstrated that the transcription level of *Ts-crt* mRNA was 1.5- and 2.3-fold higher in the ML and adult worms of *T. spiralis*, respectively, relative to that in the NBL after being normalized by GAPDH (Figure 3B).

To detect the distribution of native *Ts*-CRT in the parasite, an IFA was performed using mouse anti-*Ts*-CRT sera in the sections of different developmental stages of the worm. The IFA results showed that *Ts*-CRT was mainly expressed on the surface of all life stages of the parasite, including adult worms, ML and NBL. Some *Ts*-CRT was also observed in the inner structures of adult and larva worms. By contrast, no visible reactivity was observed in the parasite when probed with normal mouse sera at the same dilution (Figure 3C). The surface distribution of *Ts*-CRT suggests that it is accessible to host immune system as a possible immunomodulator.

### *Ts*-CRT Binds to Human C1q

**Figure 4:**
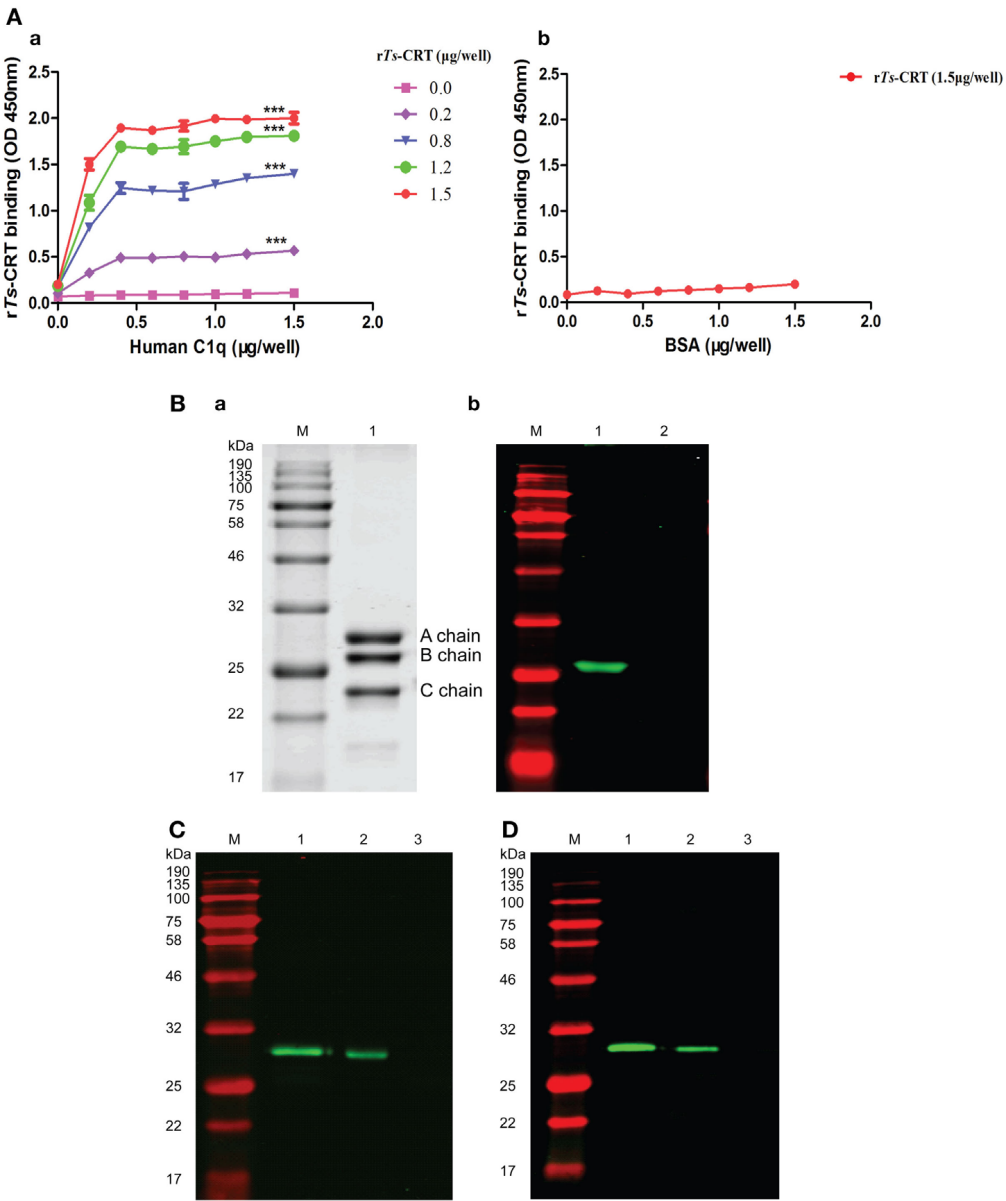
*Trichinella spiralis* calreticulin (*Ts*-CRT) binds to human C1q. **(A)** ELISA plate coated with different doses of C1q or BSA (0, 0.2, 0.4, 0.6, 0.8, 1.0, 1.2, and 1.5 µg/well), then incubated with different concentrations of recombinant *Ts*-CRT (r*Ts*-CRT) (0, 0.2, 0.8, 1.2, and 1.5 µg/well for the C1q-coated plate and 1.5 µg/well for the BSA-coated plate), and detected by anti-His antibody, demonstrating the dose-dependent binding of r*Ts*-CRT to C1q (a) but not to BSA (b). Data are shown as the mean ± SDs for three independent experiments (***p < 0.001 compared to the plate without the addition of r*Ts*-CRT). **(B)** Western blot showing that r*Ts*-CRT specifically bound to the B chain of human C1q when the C1q chains were denatured and separated. Five micrograms of C1q were run on SDS-PAGE under reducing condition, with Coomassie staining revealing the A, B, and C chains (a). Five micrograms of C1q (Lane 1) or BSA (Lane 2) were transferred to a nitrocellulose membrane, and incubated with r*Ts*-CRT (5 µg/mL), then probed with anti-His antibody (1:5,000) (b). **(C)** The human C1q complex was pulled down by r*Ts*-CRT. r*Ts*-CRT was incubated with anti-His antibody, ProteinG MicroBeads and human C1q. The eluted complexes were separated by SDS-PAGE, transferred to a nitrocellulose membrane and detected by rabbit anti-C1qA antibody. Lane 1, human C1q alone; Lane 2, r*Ts*-CRT + anti-His + C1q; Lane 3, anti-His + C1q. **(D)** The human C1q complex was pulled down by the native *Ts*-CRT in muscle larvae extracts detected by rabbit anti-C1qA antibody: Lane 1, human C1q;Lane 2, ML extracts + anti-*Ts*-CRT + C1q; Lane 3, anti-*Ts*-CRT + C1q. M, molecular weight marker.

Recombinant *Ts*-CRT or native *Ts*-CRT binding to human C1q was confirmed by different immunological assays in this study. ELISA with C1q-coated plates demonstrated that r*Ts*-CRT bound to C1q in a dose-dependent manner. When the plate was coated with 0.4 µg/well of C1q, it saturated the binding of r*Ts*-CRT up to 1.5 µg/well (Figure 4A, a). There was no obvious binding of r*Ts*-CRT to the BSA coated on plate when r*Ts*-CRT was added to 1.5 µg/well (Figure 4A, b). SDS-PAGE results revealed that C1q was separated into A, B, and C chains under reducing condition (Figure 4B, a). The far Western blot experiments revealed that r*Ts*-CRT only bound to the B chain of C1q, but not to BSA when incubated with the same concentration of r*Ts*-CRT (Figure 4B, b). To confirm whether r*Ts*-CRT or the native form of *Ts*-CRT bound to human C1q in the non-denatured condition, the natural form of C1q (complex with A, B, and C chains) was pulled down by r*Ts*-CRT bound to anti-His antibody (Figure 4C) or native *Ts*-CRT (in the ML extracts) bound to mouse anti-*Ts*-CRT antibody (Figure 4D) immobilized on ProteinG MicroBeads. Anti-His antibody or anti-*Ts*-CRT sera alone without *Ts*-CRT could not pull down C1q. These results confirmed that either r*Ts*-CRT or native *Ts*-CRT enables to bind to human C1q in their natural forms.

### r*Ts*-CRT Inhibited the Activation of Classical Complement Pathway and Hemolysis

**Figure 5:**
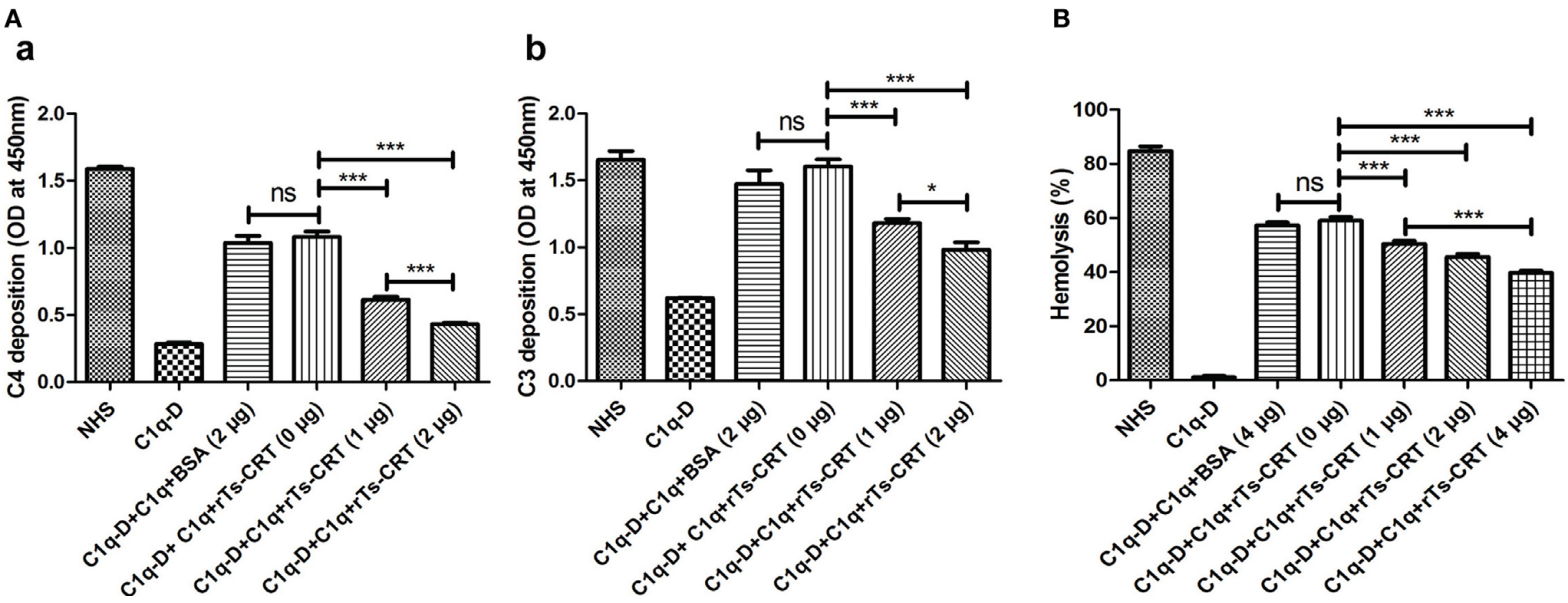
Inhibition of complement classical pathway-mediated C4/C3 generation and hemolysis by recombinant *Trichinella spiralis* calreticulin (r*Ts*-CRT). **(A)** C1q (2 µg) was incubated with different amounts of r*Ts*-CRT (0, 1, or 2 µg) or BSA (2 µg) before being added to a human IgM-coated plate (2 µg/mL). The IgM-activated classical pathway was initiated when C1q-D serum was supplemented, and the generated C3 and C4 were detected by anti-C4 and anti-C3 antibodies. The generation of C4 (a) and C3 (b) was decreased when C1q was incubated with r*Ts*-CRT in a dose-dependent manner, while normal human serum had complete activation and C1q-D alone had no activation. The BSA control exhibited no inhibition on C1q-initiated classical pathway. **(B)** Sheep red blood cells (SRBCs) hemolysis in the presence of anti-SRBC antibody was inhibited when C1q (2 µg) was incubated with different amounts of r*Ts*-CRT (0, 1, 2, or 4 µg). The OD of the supernatants was measured at 412 nm, and the percentage of hemolysis was calculated with reference to complete lysis in water. Data are shown as the mean ± SDs for three independent experiments, each experiment was done in triplicates. *p < 0.05 and *** *p* < 0.001. ns = no significant difference.

C3 and C4 depositions were used to evaluate the IgM-activated classical complement pathway. C1q was incubated with different amounts of r*Ts*-CRT before addition to IgM-coated plates. The classical complement pathway activation was completed by adding with C1q-D and was detected by measuring the resultant C3 or C4 deposited in the plate. The ELISA results showed that the IgM-activated C1q and the following cascade of classical complement pathway when C1q-D was supplemented at a similar level to NHS. However, the activation characterized by the deposition of C3 or C4 was significantly inhibited when C1q was bound by r*Ts*-CRT in a dose-dependent manner (Figure 5A). Control BSA had no effect on complement activity.

A C1q-dependent hemolytic assay was also used to determine whether r*Ts*-CRT binding to C1q would inhibit C1q-dependent classical pathway that lyses sheep red blood cells (SRBCs) preincubated with rabbit anti-SRBC antibody. As shown in Figure 5B, the supplementation of C1q in C1q-D serum initiated 60% hemolysis of the SRBCs in the presence of anti-SRBC antibody; however, the hemolysis was significantly inhibited when C1q was incubated with different amounts of r*Ts*-CRT before being added to the hemolytic system, and the inhibition was r*Ts*-CRT dose-dependent. Control BSA had no inhibitory effect, indicating that the inhibition was r*Ts*-CRT specific. C1q-D alone caused no significant hemolysis since the classical pathway could not be activated in the absence of C1q. These results confirm that r*Ts*-CRT inhibits the activation of classical complement pathway by binding to C1q.

### r*Ts*-CRT-Inhibited C1q Binding to Macrophages, C1q-Induced Monocytes/Macrophages Chemotaxis, and ROIs Release

**Figure 6:**
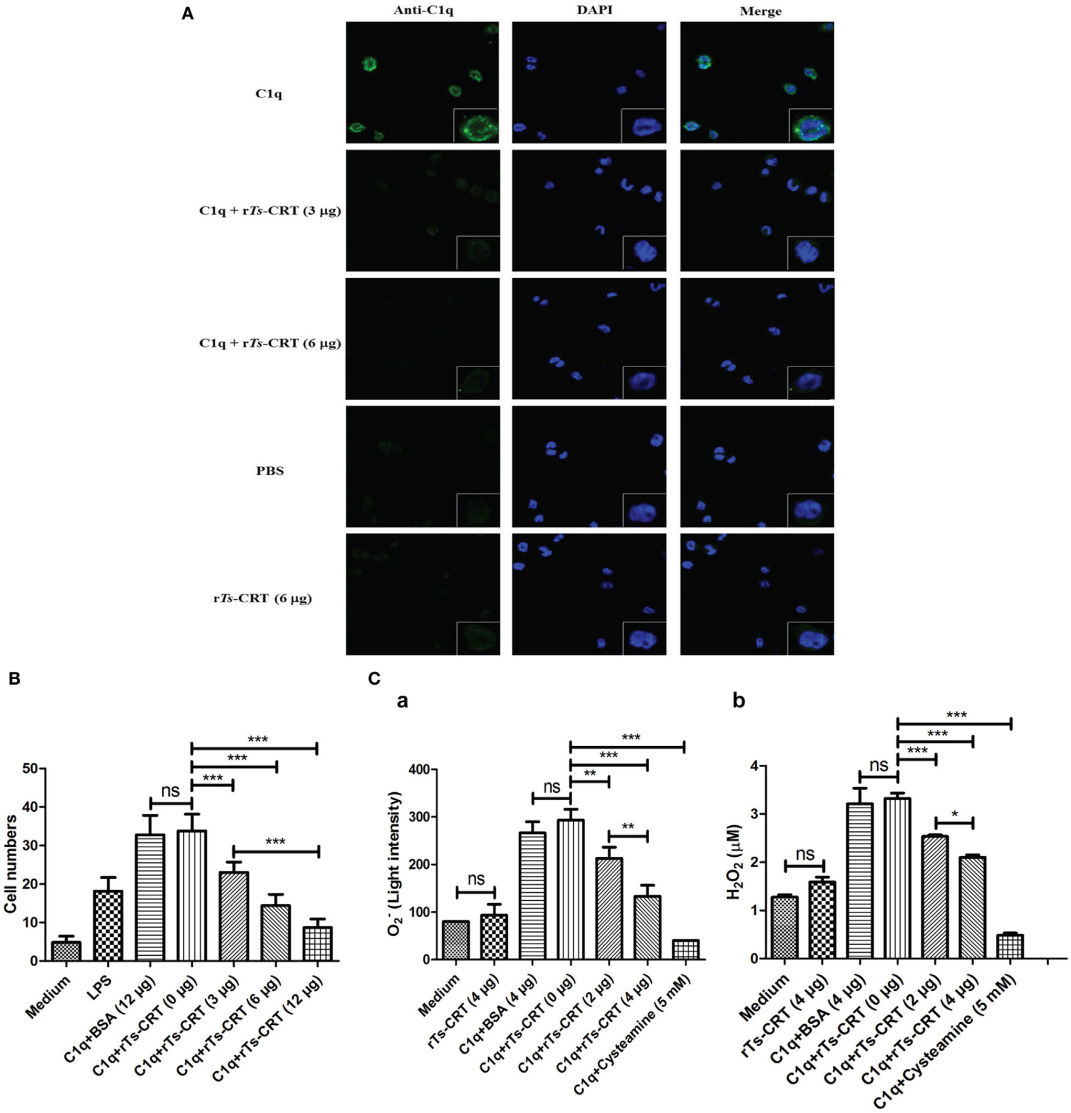
Recombinant *Trichinella spiralis* calreticulin (r*Ts*-CRT) inhibited C1q binding to macrophages and C1q-induced chemotaxis and reactive oxygen intermediates release of macrophages. **(A)** The binding of C1q on M2 type macrophages was detected by anti-C1q mAb and DyLight 488-conjugated anti-Rat IgG (green). The C1q binding on the macrophages was significantly inhibited when C1q was preincubated with different amounts of r*Ts*-CRT (0, 3, or 6 µg). Phosphate-buffered saline and r*Ts*-CRT alone served as controls. The nuclei were stained with DAPI (blue). The magnification of the photographs is 400×, and the cells at the bottom right corner are magnified at 1,000×. **(B)** The inhibition of r*Ts*-CRT on C1q-induced chemotaxis to macrophages was performed in a transwell-24-well plate. In total, 2 × 10^5^ of M2-type macrophages were seeded into the upper chamber, and the stimuli: LPS (100 ng/mL), C1q (10 nM) alone or incubated with different amounts of r*Ts*-CRT (0, 3, 6, or 12 µg) were added into the lower chamber. The number of cells traversing the membrane from 10 randomly selected fields was counted under a microscope. **(C)** The inhibition of r*Ts*-CRT on C1q-induced release of reactive oxygen intermediates (ROIs) was performed in 5 × 10^4^ THP-1 cells/well incubated with the following different stimuli: C1q (1 µg/well) alone or preincubated with different amounts of r*Ts*-CRT (0, 2, or 4 µg) or cysteamine, an inhibitor of ROIs. The generation of ${\text{O}}_{2}^{-}$ (a) and H_2_O_2_ (b) was measured in the supernatants. Data are shown as the mean ± SDs for three independent experiments, each experiment was done in triplicates. *p < 0.05, **p < 0.01, and ***p < 0.001. ns = no significant difference.

To investigate whether r*Ts*-CRT inhibits the binding of C1q to macrophages, different amounts of r*Ts*-CRT (0, 30, or 60 µg/mL) were incubated with C1q (80 µg/mL) before being added into macrophages induced from THP-1 cells by PMA and human IL-4 (33, 36, 41). The C1q binding on the macrophages was detected by IFA with anti-C1q antibody. The results showed that r*Ts*-CRT inhibited the binding of C1q to macrophages in a dose-dependent manner. PBS and r*Ts*-CRT alone without C1q yielded no obvious fluorescence detected on the cells (Figure 6A). Thus, the results suggested that r*Ts*-CRT-bound C1q and blocked C1q’s binding to macrophages.

To assess whether r*Ts*-CRT affected C1q-induced chemotaxis of macrophages, a transwell migration assay was performed. Both LPS and C1q were able to significantly attract THP-1-derived M2 macrophages migration through the membrane (Figure 6B). However, the C1q-induced macrophages migration was markedly inhibited after being incubated with different amounts of r*Ts*-CRT (0, 3, 6, or 12 µg) in a dose-dependent manner (*p* < 0.001 compared to C1q without r*Ts*-CRT). There was no effect observed for BSA when it was added up to 12 µg.

C1q binds to the C1q receptor on monocyte–macrophages to trigger the release of ROIs including ${\text{O}}_{2}^{-}$ and H_2_O_2_ (15). The incubation of C1q with r*Ts*-CRT significantly inhibited C1q stimulation on THP-1-derived monocyte–macrophages to release ${\text{O}}_{2}^{-}$ (Figure 6C, a) or H_2_O_2_ (Figure 6C, b). The inhibition of r*Ts*-CRT was dose dependent. No obvious inhibition was measured in the group incubated with BSA (4 µg), whereas cysteamine, an inhibitor of ROIs, markedly inhibited C1q-induced ROIs generation. These results further confirm that r*Ts*-CRT not only inhibits C1q binding to the receptor on monocyte–macrophages but also functionally inhibits C1q-binding triggered release of ROIs.

### Surface-Expressed *Ts*-CRT Protects NBL from Being Attacked by Host C1q-Mediated Monocyte–Macrophages

**Figure 7:**
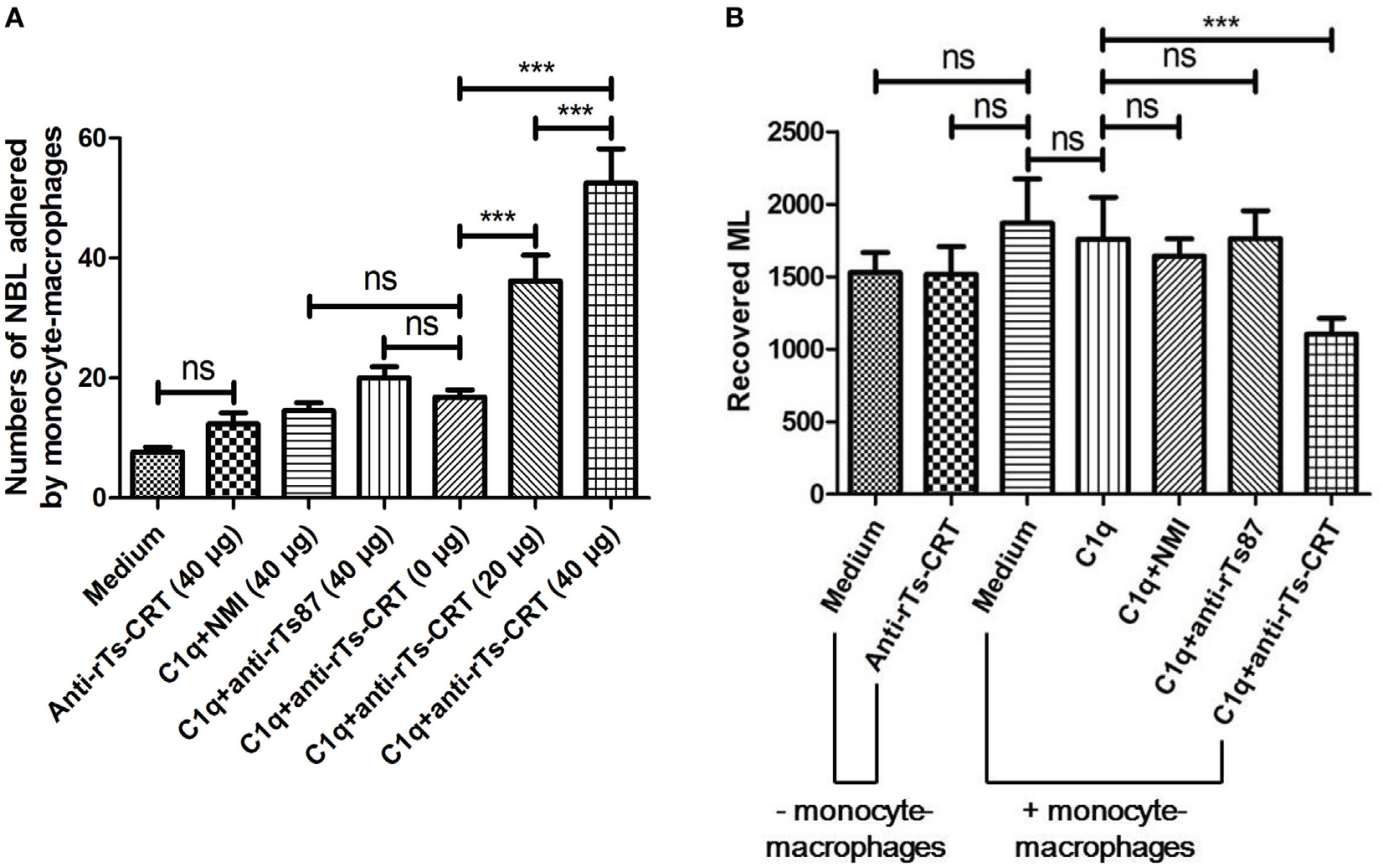
Blocking *Trichinella spiralis* calreticulin (*Ts*-CRT) on the surface of *T. spiralis* newborn larvae (NBL) enhanced the adherence of C1q-mediated monocyte–macrophages and reduced the infectivity of treated NBL *in vivo*. **(A)** NBL were preincubated with different amounts of anti-*Ts*-CRT IgG (0, 20, or 40 µg), then added to Fc receptor-blocked THP-1-derived monocyte–macrophages in the presence of C1q. NMI and anti-*Ts*87 IgG were used as controls. The worms attached with more than three cells were scored as positive. Experiments were run in triplicate. **(B)** The infectivity of NBL treated with anti-*Ts*-CRT/C1q/monocyte–macrophages was measured by passive transfer to normal mice. The ML recovered from the treated NBL was enumerated 26 days after injection. Data are shown as the mean ± SDs for three independent experiments, each experiment was done in triplicates. ***p < 0.001. ns = no significant difference.

To determine whether *Ts*-CRT expressed on the surface of parasite interferes with C1q-induced monocyte–macrophages adherence and subsequent killing of the parasite, mouse anti-*Ts*-CRT IgG was used to block native *Ts*-CRT expressed on the NBL before monocyte–macrophages being added in the presence of C1q. As shown in Figure 7A, after being blocked with anti-*Ts*-CRT IgG, the number of NBL adhered by C1q-mediated monocyte–macrophages was significantly enhanced in an antibody dose-dependent manner compared to those incubated with NMI. Incubation with mouse anti-*Ts*87, another *T. spiralis* protein located on the surface of worms (39, 40), did not significantly elevate C1q-mediated monocyte–macrophages adherence.

To further determine the infectivity of the NBL treated with anti-*Ts*-CRT/C1q/monocyte–macrophages as described above, the treated NBL were passively transferred into normal mice, and ML were collected from the muscles of infected mice 26 days after transfer. The necropsy results showed that the number of ML developing from NBL treated with anti-*Ts*-CRT/C1q/monocyte–macrophages was markedly decreased compared to that of NBL treated with C1q without anti-*Ts*-CRT (Figure 7B). No significant ML reduction was observed for NBL treated with NMI or anti-*Ts*87 combining with C1q/monocyte–macrophages. The monocyte–macrophages-involved antibody-dependent cell-mediated cytotoxicity (ADCC) was blocked by adding human Fc receptor-binding inhibitor. These results suggest that *Ts*-CRT expressed on the surface of *T. spiralis* may protect the parasite from being attacked by host C1q-stimulated monocyte–macrophages through directly binding and neutralizing the C1q function.

## Discussion

Nematode parasites are multicellular pathogens that produce many macromolecules that are secreted out of parasite or expressed on the surface to interact with host immune system as an evasion strategy to survive within host (42–44). Complement is viewed as the initial defender for pathogen clearance. By virtue of the essential role in the immune defense against invading pathogens, blocking or diverting host complement attack becomes the pathogens’ first target to employ immune evasion tactics during evolution (45–47). Many pathogens appear to share similar strategies to avert complement attack (6).

Calreticulin is a well-conserved Ca^2+^-binding protein with multiple biofunctions (23). Sequence alignment shows that *Ts*-CRT shares 43–59% identity with CRTs from other parasites, including *Trypanosoma carassii* (22), *B. malayi* (21), *N. americanus* (20), and *H. contortus* (25), indicating their conserved functions for the parasite’s survival in host. All of these CRTs have been identified in the secreted form or as expressed on the parasite surface, and they are involved in host immune regulation by binding to C1q. Moreover, there is growing evidence supporting that parasitic CRT plays important roles in regulation of adaptive immune responses in diverse parasitic disease (48). For example, *Tenia solium* CRT has been shown to elicit a TH2 response characterized by the induction of IL-10 during the parasite infection (49), which might contribute to the immune escape strategy developed by *T. solium* to suppress host immune response. The *E. coli*-expressed soluble recombinant *Ts*-CRT was stained blue by Stains-all, indicating its calcium-binding feature. Stage-specific expression analysis revealed that *Ts*-CRT could be expressed in all stages of *T. spiralis* including NBL, ML and adult worms, with a higher expression level in ML and adult worms than in NBL, indicating its importance in the parasite development and survival evolution within host. Immunolocalization with anti-*Ts*-CRT antibody demonstrated that native *Ts*-CRT was mostly expressed on the parasite surface and was also present in the excretory–secretory (ES) products of ML and adult worms, suggesting its importance as an interface between parasite and host interactions and immunological accessibility if being developed as a vaccine. Functional analysis identified that *Ts*-CRT, either as recombinant protein or native protein expressed by the parasite, was able to strongly bind to human complement C1q, more specifically to the B chain of C1q complex, indicating its modulating functions with complement activation. Indeed, we demonstrated that *Ts*-CRT binding to C1q significantly inhibited C1q-initiated complement classical activation pathway characterized by the decreased generation of C3 and C4, intermediators of classical activation initiated by human IgM, and reduced hemolytic activity on antibody-sensitized sheep blood cells, the final formation of MAC on erythrocyte membrane as a result of complement activation. The results demonstrated that *T. spiralis*-expressed *Ts*-CRT plays important roles in interfering with classical complement activation by directly binding to C1q.

C1q is a versatile pattern recognition molecule recognizing various structures and ligands and executing diverse biological functions in addition to initiating the classical complement pathway (12). Both the complement and non-complement-dependent functions of C1q play vital roles in modulating host immune response. To identify whether *Ts*-CRT binding to C1q would interfere with other C1q-activated cellular immune responses, in this study, we explored the impact of *Ts*-CRT binding to C1q on the C1q-involved macrophage activation except for the inhibition on the C1q-initiated complement classical activation pathway. It is established that monocytes and macrophages not only are important innate immune cells directly involved or associating with neutrophils and complement (50) in the clearance of invaded pathogens but also play crucial roles in TH1- and TH2-mediated adaptive immune responses (51). Even though TH2 response is generally generated in helminth infections, a mixed TH1/TH2 response was observed in human chronic trichinellosis (52). Monocytes and macrophages express some complement receptors, such as complement receptors-1 (CR1), on their surface to interact with complement components (41, 53, 54). C1q has been demonstrated to promote M2 macrophages polarization (55), M2 macrophages are involved in the TH2 immune response (56) by directly binding to C1q receptors such as CR1 and acts as an attractant to stimulate macrophages migration to infectious or inflammatory regions, indicating that C1q may play roles in tissue injury and repair (57) as well as clearance of pathogens (58). Using IFA, we confirmed that C1q bond on the surface of THP-1-derived M2-like macrophages through C1q receptors that could be inhibited by the addition of r*Ts*-CRT, indicating that r*Ts*-CRT protein could bind to C1q and block the subsequent binding of C1q to the surface of macrophages. The *Ts*-CRT-mediated inhibition of the C1q-involved macrophage functions was also reflected by the suppression of C1q-stimulated chemotaxis of macrophages and reduced their release of ROIs that are directly involved in the killing and clearing invaded pathogens (15, 16, 33, 59–61). Blocking *Ts*-CRT expressed on the surface of NBL of *T. spiralis* using anti-*Ts*-CRT antibody enhanced the adherence of C1q-activated monocyte−macrophages to the parasite larvae and reduced larvae infectivity after being transferred back to normal mice, further suggesting the protective function of parasite-expressed *Ts*-CRT against macrophage attack, possibly through binding to C1q, in addition to inhibiting complement classical activation. The antigen-antibody complex on the surface of parasite could also activate the macrophage through the immunoglobulin Fc domain binding to FcγRI and FcγRII receptors on monocyte–macrophage cells (37), however, it has been excluded by the addition of human FcγR-binding inhibitor in this study.

All the above results suggest that *T. spiralis*-expressed *Ts*-CRT plays important roles in immune evasion and survival in host, mostly by directly binding to host complement C1q, which not only reduces C1q-mediated activation of classical complement pathway but also inhibits the C1q-induced non-complement activation of macrophages. Therefore, *Ts*-CRT is an important target for vaccine or therapeutic drug development. In an attempt to further understand the interaction of *Ts*-CRT with human complement C1q at the structural and functional level, the crystal structure of *Ts*-CRT and the C1q-binding region on *Ts*-CRT, as well as other possible roles of *Ts*-CRT in host–parasite interactions, are under investigation.

## Ethics Statement

This study was carried out in accordance with the recommendations of “IRB of Capital Medical University” with written informed consent from all subjects. All subjects gave written informed consent in accordance with the Declaration of Helsinki. The protocol was approved by the “IRB of Capital Medical University” (approval number: 2016SY01). This study was carried out in accordance with the recommendations of “NIH Guidelines for the Care and Use of Laboratory Animals, Capital Medical University Animal Care and Use Committee.” The protocol was approved by the “Capital Medical University Animal Care and Use Committee” (approval number: 2012-X-108).

## Author Contributions

XZ and LZ conceived and designed the experiments. LZ, SS, YC, RS, and JH performed the experiments. XZ, LZ, BZ, and XS analyzed the data. LZ, XZ, and BZ wrote the paper. All authors reviewed the manuscript.

## Conflict of Interest Statement

The authors declare that the research was conducted in the absence of any commercial or financial relationships that could be construed as a potential conflict of interest.

## References

[1] Pozio E. World distribution of *Trichinella* spp. infections in animals and humans. Vet Parasitol (2007) 149:3–21. 10.1016/j.vetpar.2007.07.002 17689195

[2] Zhu X Su C Human Parasitology. 8th ed Beijing: People’s Medical Publishing House (2013). 286 p.

[3] Dupouy-Camet J. Trichinellosis: a worldwide zoonosis. Vet Parasitol (2000) 93:191–200. 10.1016/S0304-4017(00)00341-1 11099837

[4] Mitreva M Jasmer DP Biology and genome of *Trichinella spiralis*. WormBook (2006) 1–21. 10.1895/wormbook.1.124.1 PMC4781409 18050431

[5] Morgan BP Marchbank KJ Longhi MP Harris CL Gallimore AM. Complement: central to innate immunity and bridging to adaptive responses. Immunol Lett (2005) 97:171–9. 10.1016/j.imlet.2004.11.010 15752555

[6] Lambris JD Ricklin D Geisbrecht BV. Complement evasion by human pathogens. Nat Rev Microbiol (2008) 6:132–42. 10.1038/nrmicro1824 18197169 PMC2814840

[7] Bonaparte RS Hair PS Banthia D Marshall DM Cunnion KM Krishna NK. Human astrovirus coat protein inhibits serum complement activation via C1, the first component of the classical pathway. J Virol (2008) 82:817–27. 10.1128/JVI.01847-07 17959658 PMC2224607

[8] Bergstrom FC Reynolds S Johnstone M Pike RN Buckle AM Kemp DJ Scabies mite inactivated serine protease paralogs inhibit the human complement system. J Immunol (2009) 182:7809–17. 10.4049/jimmunol.0804205 19494305

[9] Agarwal V Sroka M Fulde M Bergmann S Riesbeck K Blom AM. Binding of *Streptococcus pneumoniae* endopeptidase O (PepO) to complement component C1q modulates the complement attack and promotes host cell adherence. J Biol Chem (2014) 289:15833–44. 10.1074/jbc.M113.530212 24739385 PMC4140937

[10] Gadjeva MG Rouseva MM Zlatarova AS Reid KB Kishore U Kojouharova MS.Interaction of human C1q with IgG and IgM: revisited. Biochemistry (2008) 47:13093–102. 10.1021/bi801131h 19006321

[11] Nayak A Ferluga J Tsolaki AG Kishore U. The non-classical functions of the classical complement pathway recognition subcomponent C1q. Immunol Lett (2010) 131:139–50. 10.1016/j.imlet.2010.03.012 20381531

[12] Nayak A Pednekar L Reid KB Kishore U. Complement and non-complement activating functions of C1q: a prototypical innate immune molecule. Innate Immun (2012) 18:350–63. 10.1177/1753425910396252 21450789

[13] Kuna P Iyer M Peerschke EI Kaplan AP Reid KB Ghebrehiwet B. Human C1q induces eosinophil migration. Clin Immunol Immunopathol (1996) 81:48–54. 10.1006/clin.1996.0156 8808641

[14] Fraser DA Laust AK Nelson EL Tenner AJ. C1q differentially modulates phagocytosis and cytokine responses during ingestion of apoptotic cells by human monocytes, macrophages, and dendritic cells. J Immunol (2009) 183:6175–85. 10.4049/jimmunol.0902232 19864605 PMC2843563

[15] Alvarez-Dominguez C Carrasco-Marin E Lopez-Mato P Leyva-Cobian F. The contribution of both oxygen and nitrogen intermediates to the intracellular killing mechanisms of C1q-opsonized *Listeria monocytogenes* by the macrophage-like IC-21 cell line. Immunology (2000) 101:83–9. 10.1046/j.1365-2567.2000.00083.x 11012757 PMC2327058

[16] Kolodziej-Sobocinska M Dvoroznakova E Dziemian E. *Trichinella spiralis*: macrophage activity and antibody response in chronic murine infection. Exp Parasitol (2006) 112:52–62. 10.1016/j.exppara.2005.09.004 16274689

[17] Zhang Z Yang J Wei J Yang Y Chen X Zhao X *Trichinella spiralis* paramyosin binds to C8 and C9 and protects the tissue-dwelling nematode from being attacked by host complement. PLoS Negl Trop Dis (2011) 5:e1225. 10.1371/journal.pntd.0001225 21750743 PMC3130009

[18] Sun R Zhao X Wang Z Yang J Zhao L Zhan B *Trichinella spiralis* paramyosin binds human complement C1q and inhibits classical complement activation. PLoS Negl Trop Dis (2015) 9:e4310. 10.1371/journal.pntd.0004310 26720603 PMC4697845

[19] Ferreira V Molina MC Valck C Rojas A Aguilar L Ramirez G Role of calreticulin from parasites in its interaction with vertebrate hosts. Mol Immunol (2004) 40:1279–91. 10.1016/j.molimm.2003.11.018 15128045

[20] Kasper G Brown A Eberl M Vallar L Kieffer N Berry C A calreticulin-like molecule from the human hookworm *Necator americanus* interacts with C1q and the cytoplasmic signalling domains of some integrins. Parasite Immunol (2001) 23:141–52. 10.1046/j.1365-3024.2001.00366.x 11240905

[21] Yadav S Gupta S Selvaraj C Doharey PK Verma A Singh SK *In silico* and *in vitro* studies on the protein-protein interactions between *Brugia malayi* immunomodulatory protein calreticulin and human C1q. PLoS One (2014) 9:e106413. 10.1371/journal.pone.0106413 25184227 PMC4153637

[22] Oladiran A Belosevic M. *Trypanosoma carassii* calreticulin binds host complement component C1q and inhibits classical complement pathway-mediated lysis. Dev Comp Immunol (2010) 34:396–405. 10.1016/j.dci.2009.11.005 19913050

[23] Michalak M Corbett EF Mesaeli N Nakamura K Opas M. Calreticulin: one protein, one gene, many functions. Biochem J (1999) 344:281–92. 10.1042/bj3440281 10567207 PMC1220642

[24] Ferreira V Valck C Sanchez G Gingras A Tzima S Molina MC The classical activation pathway of the human complement system is specifically inhibited by calreticulin from *Trypanosoma cruzi*. J Immunol (2004) 172:3042–50. 10.4049/jimmunol.172.5.3042 14978109

[25] Suchitra S Joshi P. Characterization of *Haemonchus contortus* calreticulin suggests its role in feeding and immune evasion by the parasite. Biochim Biophys Acta (2005) 1722:293–303. 10.1016/j.bbagen.2004.12.020 15716049

[26] Bi K Yang J Wang L Gu Y Zhan B Zhu X Partially protective immunity induced by a 20 kDa protein secreted by *Trichinella spiralis* stichocytes. PLoS One (2015) 10:e136189 10.1371/journal.pone.0136189 PMC4545582 26288365

[27] Gamble HR Bessonov AS Cuperlovic K Gajadhar AA van Knapen F Noeckler K International Commission on Trichinellosis: recommendations on methods for the control of *Trichinella* in domestic and wild animals intended for human consumption. Vet Parasitol (2000) 93:393–408. 10.1016/S0304-4017(00)00354-X 11099850

[28] Auxiliadora DM Bolas-Fernandez F Dynamics of the IgG3 responses following immunisation of BALB/c mice with somatic and excretory/secretory antigens from various *Trichinella* species. Folia Parasitol (Praha) (2000) 47:172–80. 10.14411/fp.2000.034 11104144

[29] Dea-Ayuela MA Rama-Iniguez S Bolas-Fernandez F. Vaccination of mice against intestinal *Trichinella spiralis* infections by oral administration of antigens microencapsulated in methacrilic acid copolymers. Vaccine (2006) 24:2772–80. 10.1016/j.vaccine.2006.01.006 16446017

[30] Yang X Yang Y Wang Y Zhan B Gu Y Cheng Y Excretory/secretory products from *Trichinella spiralis* adult worms ameliorate DSS-induced colitis in mice. PLoS One (2014) 9:e96454. 10.1371/journal.pone.0096454 24788117 PMC4008629

[31] Campbell KP MacLennan DH Jorgensen AO Staining of the Ca^2+^-binding proteins, calsequestrin, calmodulin, troponin C, and S-100, with the cationic carbocyanine dye “Stains-all”. J Biol Chem (1983) 258:11267–73. 6193121

[32] Livak KJ Schmittgen TD. Analysis of relative gene expression data using real-time quantitative PCR and the 2(-Delta Delta C(T)) method. Methods (2001) 25:402–8. 10.1006/meth.2001.1262 11846609

[33] Chanput W Mes JJ Wichers HJ. THP-1 cell line: an in vitro cell model for immune modulation approach. Int Immunopharmacol (2014) 23:37–45. 10.1016/j.intimp.2014.08.002 25130606

[34] Meng F Li C Li W Gao Z Guo K Song S. Interaction between pancreatic cancer cells and tumor-associated macrophages promotes the invasion of pancreatic cancer cells and the differentiation and migration of macrophages. IUBMB Life (2014) 66:835–46. 10.1002/iub.1336 25557640

[35] Tsuchiya S Yamabe M Yamaguchi Y Kobayashi Y Konno T Tada K Establishment and characterization of a human acute monocytic leukemia cell line (THP-1). Int J Cancer (1980) 26:171–6. 10.1002/ijc.2910260208 6970727

[36] Moulds JM Nickells MW Moulds JJ Brown MC Atkinson JP. The C3b/C4b receptor is recognized by the Knops, McCoy, Swain-langley, and York blood group antisera. J Exp Med (1991) 173:1159–63. 10.1084/jem.173.5.1159 1708809 PMC2118866

[37] Fleit HB Kobasiuk CD. The human monocyte-like cell line THP-1 expresses Fc gamma RI and Fc gamma RII. J Leukoc Biol (1991) 49:556–65. 1709200 10.1002/jlb.49.6.556

[38] Yang J Pan W Sun X Zhao X Yuan G Sun Q Immunoproteomic profile of *Trichinella spiralis* adult worm proteins recognized by early infection sera. Parasit Vectors (2015) 8:20. 10.1186/s13071-015-0641-8 25582511 PMC4299305

[39] Yang Y Zhang Z Yang J Chen X Cui S Zhu X. Oral vaccination with Ts87 DNA vaccine delivered by attenuated *Salmonella typhimurium* elicits a protective immune response against *Trichinella spiralis* larval challenge. Vaccine (2010) 28:2735–42. 10.1016/j.vaccine.2010.01.026 20105428

[40] Yang Y Zhu X Yang J Lei L Jing P Immunohistochemical localization of Ts87 antigen of *Trichinella spiralis*. J Capital Univ Med Sci (2003) 24:108–10. 10.3969/j.issn.1006-7795.2003.02.005

[41] Bohlson SS O’Conner SD Hulsebus HJ Ho MM Fraser DA. Complement, C1q, and C1q-related molecules regulate macrophage polarization. Front Immunol (2014) 5:402. 10.3389/fimmu.2014.00402 25191325 PMC4139736

[42] Dainichi T Maekawa Y Ishii K Zhang T Nashed BF Sakai T Nippocystatin, a cysteine protease inhibitor from *Nippostrongylus brasiliensis*, inhibits antigen processing and modulates antigen-specific immune response. Infect Immun (2001) 69:7380–6. 10.1128/IAI.69.12.7380-7386.2001 11705911 PMC98825

[43] Prowse RK Chaplin P Robinson HC Spithill TW. *Fasciola hepatica* cathepsin L suppresses sheep lymphocyte proliferation *in vitro* and modulates surface CD4 expression on human and ovine T cells. Parasite Immunol (2002) 2:57–66. 10.1046/j.0141-9838.2001.00438.x 11874560

[44] Guillou F Roger E Mone Y Rognon A Grunau C Theron A Excretory-secretory proteome of larval *Schistosoma mansoni* and *Echinostoma caproni*, two parasites of *Biomphalaria glabrata*. Mol Biochem Parasitol (2007) 155:45–56. 10.1016/j.molbiopara.2007.05.009 17606306

[45] Garcia BL Zhi H Wager B Hook M Skare JT. *Borrelia burgdorferi* BBK32 inhibits the classical pathway by blocking activation of the C1 complement complex. PLoS Pathog (2016) 12:e1005404. 10.1371/journal.ppat.1005404 26808924 PMC4725857

[46] Zhang J Li G Liu X Wang Z Liu W Ye X. Influenza A virus M1 blocks the classical complement pathway through interacting with C1qA. J Gen Virol (2009) 90:2751–8. 10.1099/vir.0.014316-0 19656971

[47] Laarman AJ Bardoel BW Ruyken M Fernie J Milder FJ van Strijp JA *Pseudomonas aeruginosa* alkaline protease blocks complement activation via the classical and lectin pathways. J Immunol (2012) 188:386–93. 10.4049/jimmunol.1102162 22131330

[48] Rzepecka J Rausch S Klotz C Schnoller C Kornprobst T Hagen J Calreticulin from the intestinal nematode *Heligmosomoides polygyrus* is a Th2-skewing protein and interacts with murine scavenger receptor-A. Mol Immunol (2009) 46:1109–19. 10.1016/j.molimm.2008.10.032 19108896

[49] Mendlovic F Cruz-Rivera M Avila G Vaughan G Flisser A Cytokine, antibody and proliferative cellular responses elicited by *Taenia solium* calreticulin upon experimental infection in hamsters. PLoS One (2015) 10:e121321 10.1371/journal.pone.0121321 PMC4374884 25811778

[50] Bonne-Annee S Kerepesi LA Hess JA O’Connell AE Lok JB Nolan TJ Human and mouse macrophages collaborate with neutrophils to kill larval *Strongyloides stercoralis*. Infect Immun (2013) 81:3346–55. 10.1128/IAI.00625-13 23798541 PMC3754234

[51] Gordon S. The role of the macrophage in immune regulation. Res Immunol (1998) 149:685–8. 10.1016/S0923-2494(99)80039-X 9851524

[52] Della BC Benagiano M De Gennaro M Gomez-Morales MA Ludovisi A D’Elios S T-cell clones in human trichinellosis: evidence for a mixed Th1/Th2 response. Parasite Immunol (2017) 39:1–6. 10.1111/pim.12412 28106258

[53] Ghebrehiwet B Hosszu KK Valentino A Ji Y Peerschke EI. Monocyte expressed macromolecular C1 and C1q receptors as molecular sensors of danger: implications in SLE. Front Immunol (2014) 5:278. 10.3389/fimmu.2014.00278 25018754 PMC4071343

[54] Duus K Hansen EW Tacnet P Frachet P Arlaud GJ Thielens NM Direct interaction between CD91 and C1q. FEBS J (2010) 277:3526–37. 10.1111/j.1742-4658.2010.07762.x 20716178

[55] Benoit ME Clarke EV Morgado P Fraser DA Tenner AJ. Complement protein C1q directs macrophage polarization and limits inflammasome activity during the uptake of apoptotic cells. J Immunol (2012) 188:5682–93. 10.4049/jimmunol.1103760 22523386 PMC3358549

[56] Cassetta L Cassol E Poli G. Macrophage polarization in health and disease. ScientificWorldJournal (2011) 11:2391–402. 10.1100/2011/213962 22194670 PMC3236674

[57] Vogel DY Heijnen PD Breur M de Vries HE Tool AT Amor S Macrophages migrate in an activation-dependent manner to chemokines involved in neuroinflammation. J Neuroinflammation (2014) 11:23. 10.1186/1742-2094-11-23 24485070 PMC3937114

[58] Alvarez-Dominguez C Carrasco-Marin E Leyva-Cobian F. Role of complement component C1q in phagocytosis of *Listeria monocytogenes* by murine macrophage-like cell lines. Infect Immun (1993) 61:3664–72. 8359889 10.1128/iai.61.9.3664-3672.1993 PMC281062

[59] Ji J Shu D Zheng M Wang J Luo C Wang Y Microbial metabolite butyrate facilitates M2 macrophage polarization and function. Sci Rep (2016) 6:24838. 10.1038/srep24838 27094081 PMC4837405

[60] Mukbel RM Patten CJ Gibson K Ghosh M Petersen C Jones DE. Macrophage killing of *Leishmania amazonensis* amastigotes requires both nitric oxide and superoxide. Am J Trop Med Hyg (2007) 76:669–75. 10.4269/ajtmh.2007.76.669 17426168

[61] Yang K Wu Y Xie H Li M Ming S Li L Macrophage-mediated inflammatory response decreases mycobacterial survival in mouse MSCs by augmenting NO production. Sci Rep (2016) 6:27326. 10.1038/srep27326 27251437 PMC4890015

